# Evaluating Posterior Cruciate Ligament Integrity in Inflammatory Arthritis Patients Prescribed Biologic Agents: A Radiological Case-Control Study

**DOI:** 10.7759/cureus.13160

**Published:** 2021-02-05

**Authors:** Patrick Nolan, Michael O'Sullivan, Áine Gorman, Stephen Eustace, Ausaf Mohammad, Eoin Sheehan

**Affiliations:** 1 Department of Orthopaedics, Midlands Regional Hospital Tullamore, Tullamore, IRL; 2 Department of Rheumatology, Midlands Regional Hospital Tullamore, Tullamore, IRL; 3 Department of Radiology, Cappagh National Orthopaedic Hospital, Dublin, IRL

**Keywords:** inflammatory arthritis, rheumatoid arthritis, tkr, total knee arthroplasty, posterior cruciate ligament, biologic agents

## Abstract

Introduction

Patients with inflammatory arthropathies present a significant challenge to the arthroplasty surgeon when they present with symptomatic degenerative changes of their knee joint. Debate is ongoing regarding the selection of implants for this cohort of patients. There is conflicting evidence for the use of posterior-stabilising (PS) over cruciate-retaining (CR) designs in this cohort. Biologics are licensed for use in moderate-to-severe disease that has not responded to conventional treatment. To our knowledge, there are no studies that have assessed the integrity of the posterior cruciate ligament (PCL) on magnetic resonance imaging (MRI) in these patients with more advanced disease prescribed biologic agents.

Aim

The aim of this study is to assess the integrity of the PCL on MRI in patients with inflammatory arthritis who are prescribed biologic agents.

Methods

A case-control study was performed, with cases identified through chart review to confirm prescription of biologic agents for inflammatory arthropathies, who also had contemporaneous MRI knee scans performed. Knee MRIs for age- and sex-matched controls with osteoarthritis (OA) and meniscal pathology were identified from the National Joint Registry and the Hospital In-Patient Enquiry (HIPE), respectively.

The MRIs were reviewed by two musculoskeletal radiologists who were blinded to the clinical details. They were asked to assess the MRIs to determine PCL integrity, synovial Inflammation, and any associated pathology.

Results

No difference was noted in the rate of synovitis, PCL attenuation, or PCL tears between the OA and inflammatory arthropathy groups (p > 0.05).

Conclusions

The results of this study show no difference in the integrity and continuity of the PCL in those patients for age- and sex-matched controls on MRI. This finding lends support to the use of CR total knee arthroplasty in patients with inflammatory arthropathy on biologic agents.

## Introduction

Inflammatory arthropathies are chronic inflammatory conditions that can result in intra-articular damage to cartilage and bone [1]. It is estimated that up to one-quarter of patients with rheumatoid arthritis (RA) will undergo total knee arthroplasty (TKA) within 21 years of disease onset [2].

These patients present a significant challenge to the arthroplasty surgeon when they present with symptomatic degenerative changes of their knee joint. Patients with inflammatory arthropathies more commonly present for TKA at a younger age [3]. Implant selection and survival therefore are paramount in these patients.

There has been much debate around the most appropriate implant in these patients. There is no consensus regarding whether cruciate-retaining (CR) or posterior-stabilising (PS) implant designs are superior in the treatment of patients with inflammatory arthritis (IA). Critics of the CR designs cite posterior instability and high revision rates as major concerns secondary to posterior cruciate ligament (PCL) insufficiency [4].

Scott et al. found that 95% of RA patients undergoing a TKA had an intact PCL [5]. Miller et al. found that survivorship of CR designs in RA patients at 10- and 17-year follow-up was excellent and found no cases where revision was due to posterior instability [6].

Evidence suggests that it is safe to use CR designs in patients with IA [6]. Biologic drugs have been in clinical practice for over 20 years in the treatment of chronic IA [7]. These drugs have had a significant impact on the clinical outcomes and natural history of these diseases [8]. Biologics are licensed for use in moderate-to-severe RA that has not responded to conventional disease-modifying anti-rheumatic drugs (DMARDs) [9]. To our knowledge, there are no studies that have assessed the integrity of the PCL in these patients with more advanced disease who are prescribed biologic agents.

The objective of this case-control study is to assess the integrity of the PCL in patients with inflammatory arthropathies prescribed biologic agents.

## Materials and methods

We used a case-control design for this study where we compared the integrity of the PCL in patients with inflammatory arthropathies with controls for osteoarthritis (OA) and meniscal pathology.

The patient population was identified from our institution, which is a tertiary referral centre. We retrospectively identified a cohort of patients with a pre-existing diagnosis of an inflammatory arthropathy identified from the Hospital In-Patient Enquiry (HIPE) department in our institution. The medical records of these patients were analysed on the Picture Archiving and Communication System (PACS).

The medical records of the patients were also analysed to determine what biological agents these patients were prescribed. To be eligible for inclusion, patients had to have a confirmed diagnosis of an inflammatory arthropathy ( RA, psoriatic arthritis, or reactive arthritis), have undergone a knee MRI, and had be taking a prescribed biologic agent for an inflammatory arthropathy. Exclusion criteria included those patients whose knee MRI imaging was inaccessible or who underwent an MRI greater than six months prior to knee replacement.

Prior to conducting the study, a power analysis was conducted to determine the number of cases required to assess a binomial outcome, in this study, whether the PCL was anatomically normal on MRI scanning or not. Assumption incorporated for this was an alpha of 0.05 and power of 80%. The anticipated incidence of inflammatory change affecting the knee of patients with an inflammatory arthropathy was placed at 65% based on recent papers including the one by Oláh et al. who state that 20-70% of rheumatoid cervical spines are affected by the inflammatory process [10]. The anticipated incidence of inflammatory change with the knee undergoing total knee replacement for OA was set at 20%. This is based on the finding of Hill et al. who showed that in knees with OA, 22.8 had complete ACL tear, with PCL tear at 0.6%, on MRI scanning [11]. Power analysis determined that 16 cases and 16 controls would be required to assess for differences between the groups.

The control group for patients with OA was identified from the Irish National Joint Registry. Patients who underwent a TKA for OA in our tertiary referral centre were identified from the Joint Registry. This cohort of patients had their charts analysed to identify patients who had previously underwent contemporaneous knee MRI. Inclusion criteria for this group included patients with a diagnosis of OA who underwent a TKA in our centre and had a knee MRI performed prior to surgery. Exclusion criteria included patients whose MRI images were inaccessible.

The second control group of patients with meniscal pathologies who had undergone a knee arthroscopy were identified from the HIPE department in our centre. Similarly, this cohort of patients then underwent a medical record review on the PACS to identify whether a knee MRI had been performed. Inclusion criteria included patients who had a confirmed diagnosis of meniscal pathology, had undergone a knee arthroscopy, and had a knee MRI performed prior to surgery.

The patient groups were matched according to age and sex. Patient demographics and categorical data are presented as descriptive statistics (Table 1). The chi-squared test was used to determine if any difference existed for categorical variables. Student’s t-test was used to examine continuous variables. A p-value of <0.05 was considered significant. All statistical analysis was conducted using SPSS Version 26 (IBM Corp., Armonk, New York, USA).

**Table 1:** Descriptive statistics for age and sex of cases and control groups

	Inflammatory Arthritis (n=16)	Osteoarthritis (n=20)	Meniscal Pathology (n=26)
Mean age	53.7	55.0	47.9
Sex			
Male	7	11	11
Female	9	9	14

Three cohorts of patients were identified, each with a diagnosis of one of the following pathologies: meniscal tear, OA, or RA. An anonymised, encrypted Microsoft Excel database was populated with medical record numbers and limited demographic information (age and sex) for each patient.

Two readers, a musculoskeletal radiology fellow and a consultant musculoskeletal radiologist, reviewed the most recent knee MRI study performed on each patient on a PACS workstation. The readers were blinded to both the imaging report and the cohort to which each patient belonged. Imaging had been performed with a 1.5T magnet (SIGNA Excite 1.5T, General Electric Medical Systems, Waukesha, Wisconsin, USA). The protocol for the knee MRI studies included axial and sagittal proton density-weighted fat-suppressed sequences, coronal T1-weighted spin-echo, and coronal fat-suppressed T2-weighted spin-echo sequences. The readers gave a consensus opinion on the following imaging features in the case of each patient: integrity of the PCL, which was graded as normal, attenuated, or torn; the presence of synovial inflammation, and any significant associated pathology which was further described if present.

## Results

Inflammatory arthritis

A total of 16 patients with IA meeting the inclusion criteria were included in our study. RA was the most common diagnosis in this cohort with 12 patients, while there were three patients with psoriatic arthritis and one patient with reactive arthritis. The age and sex characteristics of this cohort are given in Table 1.

All patients included in our study were prescribed biologic agents for the treatment of IA. The mean duration from the diagnosis of IA to the MRI knee being performed was 5.2 years. The mean duration from the commencement of a biologic agent to the MRI knee being performed in our study was 2.5 years.

Osteoarthritis

A total of 20 patients with a diagnosis of OA were included in our study.

Meniscal pathology

A total of 26 patients with meniscal pathology confirmed on an MRI scan were included in our study.

The patients were matched according to age and sex, and there was no statistical difference between the groups under any analysis.

MRI findings

The outcomes that we measured were the integrity of the PCL, which was graded as normal, attenuated, or torn; the presence of synovial inflammation, and any significant associated pathology which was further described if present. There were several statistically significant findings identified following a review of all patients’ imaging.

Inflammatory arthritis versus meniscal pathology

There was more synovial inflammation noted in the IA cohort in comparison to the meniscal pathology cohort. This was found to be statistically significant (p < 0.05), and all other variables were equal.

Inflammatory arthritis versus osteoarthritis

There was more PCL attenuation noted in the OA cohort in comparison to the IA cohort. This was found to be statistically significant (p = 0.03). All other variables were equal on analysis.

Inflammatory arthritis versus osteoarthritis versus meniscal pathology

There was no significant difference noted between groups for the outcomes measured such as normal PCL (p = 0.055), torn PCL (p = 0.148), and PCL attenuation (p = 0.055). There was a significant difference noted for synovial inflammation (p = 0.017); however, a Bonferroni correction was performed to assess this further and was found not to be significant.

We are therefore unable to reject the null hypothesis that the PCL of patients on biologic agents for IA is the same as those patients with OA and meniscal pathology on MRI scanning.

## Discussion

Patients with IA present significant challenges to the arthroplasty surgeon when they present with symptomatic degenerative changes to the knee joint. Implant selection in these patients remains a controversial topic, and many surgeons elect to utilise a PS implant over CR implants.

Laskin and O’Flynn in 1993 found high rates of posterior instability and revision surgery in patients with RA who had undergone TKA utilising CR designs [4]. These patients were then subsequently found to have PCL insufficiency [4]. This naturally led to concerns surrounding the use of CR designs in patients with IA.

Miller et al. studied the long-term results of CR designs with up to 25-year follow-up in patients with RA [6]. The authors concluded that posterior instability due to PCL insufficiency is rarely the cause for revision in these patients [6]. Lee et al. demonstrated excellent long-term survivorship at a minimum of 15-year follow-up with CR designs in RA patients and found no cases of revision resulting from PCL insufficiency [12].

Li et al. performed a meta-analysis which concluded that CR and PS designs in TKA have similar clinical outcomes [13]. Some studies have even suggested improved long-term survivorship with CR designs [14]. Advocates of CR designs in TKA cite long-term survivorship in addition to preservation of bone stock and controlled femoral rollback as advantages of this design over PS designs [15].

Biologic agents are licensed for use in moderate-to-severe RA that has not responded to conventional DMARDs [9]. Prescription of biologic agents can therefore indicate more advanced disease. Our study is the first to evaluate the integrity of the PCL in IA patients prescribed biologic agents using MRI.

More synovial inflammation was noted in the IA cohort versus the meniscal pathology cohort in our study (p < 0.05). This is an expected finding with synovial inflammation a recognised component of IA [16].

More PCL attenuation was noted in the OA cohort than the IA cohort in our study (p = 0.033). There are no data in the literature to explain this finding. Hill et al. found a PCL rupture rate of 0.6% in OA patients [11]. However, there are limited data on PCL attenuation in OA and IA patients. This finding may be explained by the use of DMARDs and biologic agents which have played a role in delaying the progression of the disease process in IA. The prescription of these drugs may explain why there is more significant PCL attenuation in the OA cohort compared to the IA cohort. Further research must be conducted, however, to explain this finding.

There was no significant difference in outcomes between groups (p-values all being >0.05). We are therefore unable to reject the null hypothesis for this study that states that the integrity of the PCL of those patients with IA on biologic agents is the same as those patients with OA and meniscal pathology on MRI scanning.

There are several limitations to this retrospective case-control study. There may be some selection bias to how the patients were chosen for this study. The sample size of patients in this study was small even, though a power analysis was conducted to determine the number of cases. Additionally, attempts were made to account for confounding factors, but confounding bias remains a possibility in this study.

## Conclusions

This study has demonstrated that there is no significant difference in the integrity of the PCL in IA patients on MRI scanning. The authors feel that this finding lends support to the use of CR designs in TKA in patients with IA. The authors conclude that further research is required in this area such as biomechanical studies to assess PCL integrity in patients prescribed biologic agents for IA. Additionally, there may be a role for a prospective blinded clinical trial that aims to correlate PCL integrity findings on MRI with histopathological findings from PCL specimens of IA patients undergoing TKA.
